# Strength gains and distinct acute blood lactate responses induced by stepwise load reduction training in healthy males

**DOI:** 10.3389/fphys.2025.1658993

**Published:** 2025-09-04

**Authors:** Zhuo Zeng, Chengyu Zhou, Wenhui Yin, Tao Chen, Te Han, Yongmin Xie, Aiguo Zhou

**Affiliations:** ^1^ School of Strength and Conditioning Training, Beijing Sport University, Beijing, China; ^2^ School of Social Sciences, Tsinghua University, Beijing, China

**Keywords:** stepwise load reduction training, medium load, resistance training, drop sets, muscle strength, blood lactate

## Abstract

**Introduction:**

This study investigated whether stepwise load reduction training (SLRT) yields comparable or superior effects to medium load resistance training (MLRT) on one-repetition maximum (1RM) barbell back squat, thigh circumference (TC), muscle endurance (ME), counter movement jump (CMJ) performance, and acute blood lactate (BL) levels.

**Methods:**

Thirty healthy, physically active males completed both the SLRT and MLRT protocols in a crossover design to assess acute blood lactate responses firstly. Then they were randomly assigned to SLRT, MLRT, or control (CON) groups using a sealed envelope method for an 8 weeks intervention. Anthropometric data were collected at baseline. Performance metrics (1RM, TC, ME, and CMJ) were measured at baseline, week 4, and post intervention. Blinding was not feasible due to the visible nature of interventions. To minimize bias, testing was conducted by staff not involved in training, with standardized warm-ups and protocols applied across groups. Training volume, frequency and assessment timing were matched between SLRT and MLRT. Participants were instructed to avoid other structured training, and adherence was monitored weekly.

**Results:**

The results showed that both SLRT and MLRT significantly improved 1RM and ME, but SLRT produced greater gains. No significant differences were observed in TC. Additionally, SLRT led to significantly better CMJ performance and higher BL levels at immediate, 4th, 7th, and 9th minutes post exercise. The CON group performed significantly worse on all long-term outcomes compared to both SLRT and MLRT. While both SLRT and MLRT effectively enhance muscle strength, SLRT yields superior improvements in 1RM, ME, CMJ performance, and acute BL accumulation under equivalent training volumes.

**Discussion:**

These results suggest that SLRT may offer enhanced anaerobic conditioning benefits and superior adaptation potential. However, the findings should be interpreted with consideration of certain limitations, including the homogeneity of the sample and the relatively short intervention duration.

## Graphical Abstract

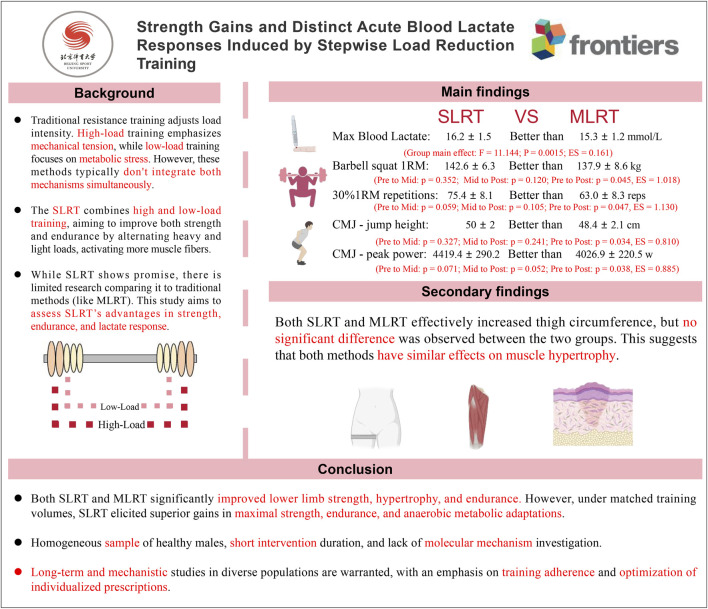

## Highlights

• Both stepwise load reduction training (SLRT) and medium load resistance training (MLRT) led to significant improvements in the 1RM and muscle endurance (30% 1RM maximum repetitions), with SLRT showing superior results compared with MLRT, particularly for the long-term increase in the 1RM.
• Compared with MLRT, SLRT resulted in significantly higher blood lactate levels at multiple time points (immediately after exercise and at the 4th, 7th, and 9th minutes), indicating greater metabolic stress and anaerobic stimulus induced by SLRT.
• SLRT significantly improved the CMJ height and peak power, highlighting its potential for enhancing explosive power and anaerobic ability. In contrast, no significant improvements were observed in CMJ performance in the MLRT or control groups.

## 1 Introduction

Resistance training (RT) is a fundamental component of physical fitness and is widely acknowledged for its effectiveness in improving muscular strength, endurance, and hypertrophy (Fleck and Kraemer, 2014; Kraemer et al., 2017). Traditional RT protocols are typically classified by load intensity and volume, with high-load and low-load training being the two most common approaches (Schoenfeld et al., 2017). High-load training emphasizes mechanical tension through the use of heavier weights and fewer repetitions, primarily aimed at developing maximal strength. In contrast, low-load training focuses on metabolic stress by utilizing lighter weights with greater repetitions and shorter rest intervals, thereby promoting muscular endurance and hypertrophy via increased lactate accumulation and muscular fatigue (Anderson and Kearney, 1982). The primary mechanism underlying these differences lies in the selective recruitment of motor units (Marshall et al., 2022). High-load RT activates higher-threshold motor units, enhancing mechanical tension, whereas low-load RT engages lower-threshold units, leading to greater metabolic stress (Henneman, 1957; Sale, 1987; Wernbom et al., 2007). However, neither approach effectively elicits both mechanical tension and metabolic stress simultaneously.

To address this limitation, researchers introduced the concept of stepwise load reduction training (SLRT), a novel method that integrates high-load and low-load RT within a single session (Ozaki et al., 2020). SLRT is designed to harness the combined benefits of both training modalities—initiate with heavy loads to induce mechanical tension, followed by lighter loads with short rest intervals to elicit metabolic stress. Previous studies have shown that this strategy can produce synergistic effects, enhancing both strength and endurance adaptations by targeting distinct physiological mechanisms (Ozaki et al., 2018). Specifically, compared with conventional single-load RT, SLRT has been reported to recruit a broader spectrum of muscle fibres and elicit higher levels of muscle activation. The alternating load structure may further improve both aerobic and anaerobic capacities by simultaneously promoting muscular strength, hypertrophy, and metabolic endurance. Despite muscle adaptation, the characteristic of gradually decreasing load in its training structure warrants further consideration. In later stages of SLRT, relatively low loads are employed, deviating from traditional high load training principles. Some studies on low-load resistance training suggest that, in the absence of artificially induced ischemia, resistance training with loads below 65% of 1RM is typically insufficient to promote substantial muscle hypertrophy (Schoenfeld, 2010). While high-repetition, low-intensity training can induce significant metabolic stress, the load used is inadequate to effectively recruit and fatigue the body’s high-threshold motor units. Additionally, SLRT presents certain operational limitations. Its structure requires practitioners to quickly change loads, which places high demands on the variety and availability of training equipment. This can hinder the fluidity and efficiency of training, especially when equipment resources are limited. To address these limitations, Nuzzo et al. proposed the connected adaptive resistance exercise (CARE) device, an intelligent apparatus that automatically adjusts the load based on the practitioner’s muscle fatigue levels (Nuzzo and Nosaka, 2022). This innovation enhances the efficiency of SLRT training and mitigates the impact of equipment limitations, thus offering greater potential for the real-world application of SLRT.

Nevertheless, despite the recognized potential of SLRT in promoting muscle adaptation, there remains a lack of sufficient evidence supporting its long-term effects. While comparisons between SLRT and low or high load training have been made, high quality randomized controlled trials comparing SLRT with medium load resistance training (MLRT) are still limited. Furthermore, whether SLRT can lead to sustained improvements in muscle strength and endurance has not been definitively confirmed. Similarly, while existing literature suggests that SLRT has potential for improving anaerobic capacity, there remains a significant gap in research regarding the acute biochemical responses following SLRT, particularly the dynamics of blood lactate (BL). As a byproduct of anaerobic glycolysis, BL serves as a critical biomarker in the metabolic stress process during resistance training (Tékus et al., 2012). Elevated lactate levels indicate an increased reliance on anaerobic energy systems, heightened metabolic stress, and are associated with increased muscle fatigue and exercise intensity. Previous studies have demonstrated that short rest intervals in resistance training (RT) can significantly increase lactate accumulation by prolonging muscle engagement and anaerobic metabolism (McKendry et al., 2016; Golas et al., 2019). In this context, SLRT shares similarities with MLRT, particularly in the metabolic stress and lactate response accumulated during training. The use of short rest intervals and alternating loads may trigger similar lactate kinetics in SLRT and MLRT. These findings offer new insights into SLRT’s potential for enhancing anaerobic capacity and promoting recovery (Kraemer et al., 1990; Gonzalez et al., 2016).

Thus, understanding the comparative efficacy of SLRT and MLRT is critical for optimizing resistance training protocols, especially for individuals aiming to enhance both strength and endurance. The present study aims to address this gap by investigating the differential effects of SLRT and MLRT on long-term adaptations in muscle strength, hypertrophy, and muscular endurance, as well as acute changes in blood lactate levels in healthy male participants. These findings may provide valuable guidance for developing more efficient and targeted resistance training strategies.

## 2 Methods

### 2.1 Participants

The sample size was preestimated via G*Power 3.1 software (Dusseldorf, Germany). We set the effect size at *f* = 0.35, with *α* = 0.05, and power (*1-β*) = 0.8. The estimation indicated that a minimum of 21 participants was required for this study. Considering a potential sample dropout rate of 20%, a minimum of 26 participants were recruited for this study. Finally, thirty healthy and active males with at least 3 years of RT experience (age: 21.78 ± 0.75 years; height: 177.12 ± 2.16 cm; weight: 75.84 ± 3.07 kg) volunteered to participate in the study (post hoc sensitivity analysis indicated that this sample size retained ≥80% power for detecting effect sizes as small as *f* = 0.27). They were instructed to avoid other physical activities and maintain their usual dietary patterns throughout the study. Past or present smokers and anyone taking any medications were excluded. All participants provided informed consent before participating in the study. The data of the subjects’ anthropometrics are shown in Table 1. We conducted the study in accordance with the Declaration of Helsinki and obtained approval from the Ethics Approval Form for Sports Science Experiments of Beijing Sport University (2024389H), China. During the course of this study, all participants successfully completed the intervention and testing as planned, with no instances of dropout. Although three participants in the control group experienced mild cold symptoms during the 8-week intervention period, these symptoms did not affect their ability to complete the tests as scheduled. Throughout the study, we closely monitored participants’ health status and took necessary measures to ensure their safety during the research.

**TABLE 1:** Subject anthropometrics.

Characteristics	SLRT	MLRT	CON	*P* (S-M)	*P* (S-C)	*P* (M-C)
Age (years)	21.30 ± 1.49	21.8 ± 1.23	22.10 ± 1.29	0.397	0.153	0.394
Heights (cm)	177.10 ± 2.60	177.40 ± 2.99	178.20 ± 2.66	0.830	0.421	0.196
Weights (kg)	75.17 ± 2.22	74.48 ± 2.08	76.24 ± 1.64	0.599	0.183	0.077
Training experience (years)	3.80 ± 0.92	3.90 ± 0.876	4.10 ± 1.20	0.758	0.576	0.693
Barbell back squat 1RM (kg)	130.40 ± 6.67	129.10 ± 7.03	128.60 ± 6.38	0.657	0.483	0.601
Thigh circumference (cm)	55.20 ± 2.2	55.03 ± 1.88	54.02 ± 1.75	0.859	0.117	0.288
30%1RM max repetitions (reps)	37.70 ± 7.07	35.80 ± 7.32	36.20 ± 5.41	0.521	0.572	0.642

*NOTE: S-M* represents the *p* value between SLRT, and MLRT; *S-C* represents the *p* value between SLRT, and CON; *M-C* represents the *p* value between MLRT, and CON; *p* > 0.05 indicates that there was no difference between the two groups.

### 2.2 Experimental design

This study employed both a randomized crossover design and a randomized parallel control design to compare the effects of stepwise load reduction training (SLRT) and medium-load resistance training (MLRT) on acute blood lactate (BL) changes, as well as long-term improvements in barbell back squat 1RM, thigh circumference, and muscle endurance (30% 1RM repetitions). Three weeks before the start of the 8-week training program, participants were required to complete a familiarization session, a pre experiment session and a BL test session consecutively. At least 72 h of washout periods were implemented between each session. The randomization process for each session requiring group assignment was completed using the sealed envelope method. During the 8-week intervention, the SLRT group performed an initial high-load (80%1RM) set followed by four drop sets at 65%, 50%, 40%, and 30% 1RM, with minimal or no rest between sets. To ensure consistency, a specially assembled barbell was used for varying loads, with plates swapped within 3 s after each set’s failure. The MLRT training program was designed on the basis of the pre experiment results. The 1RM, TC, ME and CMJ were measured at the familiarization session, after 4 weeks and at the end of the intervention.

### 2.3 Familiarization session

During the familiarization session, participants were introduced to the evaluation process for all test tasks and indicators to reduce learning effects and physical discomfort during the formal experiment. At the start of the familiarization session, the testing tools were briefly examined, and the participants were briefed on test precautions: 1) avoid high-intensity physical exercise, consume caffeine- or alcohol-containing beverages for 24 h prior to the experiment, and ensure at least 8 h of sleep; 2) hydrate appropriately and refrain from eating for 2 h before the test; 3) attempt to schedule tests at the same time, ideally a deviation of 1 h; 4) maintain consistent or similar training; and 5) refrain from engaging in high-intensity training activities during the washout period. On the first day of the first week, the participants were introduced to the testing procedures, which included methods for the BL test, the barbell back squat 1RM test, the thigh circumference test and the 30%1RM maximum repetitions test. Additionally, testers need to collect and record subjects’ age, height, weight, years of training and CMJ performance. On the second day, the participants were placed in the thigh circumference and barbell back squat 1RM. After a 72-h washout period, participants underwent the 30% 1RM maximum repetition test.

#### 2.3.1 One-repetition maximum barbell back squat test

The 1RM test used a barbell back squat, following the National Strength and Conditioning Association (NSCA) guidelines. During this test, participants were requested to position their feet slightly wider than their shoulder width, rotate their toes outwards, and descend until their thighs were parallel to the floor. All the participants performed the following warm-up sets: 6 reps at 40%, 4 reps at 60%, and 2 reps at 80% of the predicted 1RM from the familiarization session. During the formal test, the participants initially attempted a weight they could squat for 5–10 repetitions, followed by a 2-min rest. If the participant could not achieve the required depth (the lower surface of the thigh was parallel to the ground) or could not return to the upright position, the attempts were deemed failure. Subsequent attempts amplified the weight by 10%–20% each time, with a 2–4 min rest between sets. The subject’s 1RM was determined within 3–5 attempts (Miller, 2012). The clinometer was used to monitor the standardization of the subjects’ barbell back squats, the professional (NSCA-CSCS professional certified personnel) was given voice prompts, and the qualified personnel monitored the site.

#### 2.3.2 Thigh circumference test

The thigh circumference test measured the participants’ advantage side thigh. During this test, participants were required to stand as wide as the shoulder and two legs bear the weight equally. The experimenter used a marker pen to mark the position of the stripe under the subject’s hip, recorded it with a photograph, and then measured the circumference of the thigh horizontally with a tape measure. The thigh circumference test was completed three times during the familiarization session. Each tester took one measurement, as there were three testers in the study, so there were three measurements in total. If the difference between the results measured each time was greater than 0.5 cm, the participant needed to be tested again (Miller, 2012).

#### 2.3.3 Maximum repetitions of the 30% 1RM barbell back squat

The requirements of the 30% 1RM barbell back squat maximum repetition test were consistent with those of the 1RM test. The participants were asked to squat at 30% 1RM until failure. In the posttest, this test still uses the value of the pretest to present the progress of participants’ muscle endurance (Ozaki et al., 2018).

#### 2.3.4 Counter movement jump test

The counter movement jump (CMJ) test reliably assesses explosive strength (McLellan et al., 2011). The CMJ jump height and peak power data were collected via the KISTLER 9286AA force platform. Under the guidance of researchers, participants performed three consecutive CMJ attempts, with 1–2 min rest intervals between each attempt, and the best score was recorded. During the CMJ, participants stood on the force platform with their hands placed on their hips, maintaining an upright posture. Then, they squatted to 90 degrees of knee flexion and exerted maximal effort to jump vertically.

### 2.4 Pre experiment session

To determine the training program for the MLRT group, ten participants were randomly selected for two rounds of pre-experiments during the second week, with a 72-h interval between the two rounds to minimize potential fatigue effects. Each round of selection was conducted through a random lottery, and 10 participants were selected per round, with the possibility of overlap between the participants in the two round. The selected participants were required to complete one session of SLRT, recording the number of squats completed in each set and the total number of squats across all 5 sets. The results of the pre-experiment are shown in Table 2 (ICC First - Second = 0.832 > 0.75), indicating good reliability. Based on the results of the pre-experiment and in accordance with the American College of Sports Medicine guidelines, 70%–85%1RM was defined as high intensity, 50%–70%1RM was defined as medium intensity, and 30%–50%1RM was defined as low intensity. Therefore, considering the equation of training volume, the MLRT group was required to complete 5 sets of medium-load (67% 1RM) resistance exercises, with 10 squats per group and 50 squats in total.

**TABLE 2:** The results of the pre experiment, which tests the average number of repetitions in the SLRT.

Test session	80%1RM	65%1RM	50%1RM	40%1RM	30%1RM	Total
First test (reps)	7.70 ± 1.89	8.80 ± 1.14	9.80 ± 1.75	11.10 ± 1.29	12.50 ± 1.84	49.90 ± 4.23
Second test (reps)	7.90 ± 1.73	8.90 ± 0.88	10.1 ± 1.29	11.20 ± 0.92	12.40 ± 1.07	50.50 ± 3.10

### 2.5 Blood lactate test session

In the third week, participants completed one SLRT intervention and one MLRT intervention at 72-h intervals. Peripheral blood (approximately 0.5 µL) was collected using a lactate analyser (lactate scout, EKF Diagnostics, Germany) (Tanner et al., 2010). The blood lactate test was conducted in a quiet state before the intervention and immediately after the 4th, 7th and 9th minutes of exercise to assess lactate levels at different time points. The highest blood lactate value observed between 0 and 9 min was recorded. Calibration was performed prior to testing, and participants’ fingertips were disinfected with alcohol before blood collection. For sampling, a disposable blood collection needle was used to puncture the participants’ sterilized fingers. The first drop of blood was discarded, and the second drop was used for testing (Moran et al., 2012).

### 2.6 Training program

Thirty participants were randomly divided into one of the following three groups: (1) the SLRT group (n = 10): a single set starting with a high-load (80% 1RM) followed by four drop sets at 65%, 50%, 40%, and 30% 1RM, with minimal or no rest between sets. Each load was performed until concentric failure, with contractions as fast as possible in the concentric phase. Concentric failure is defined as when the participant is unable to perform a repetition with their own strength or when there is a noticeable change in movement mechanics during the repetition. This judgment is made by supervisors with CSCS certification to ensure consistent and accurate assessment. No specific repetition limit was set for each load stage, and the session ended after all load stages were completed. To ensure consistency, a specially assembled barbell was used for varying loads, with plates swapped within 3 s after each set’s failure. (2) the MLRT group (n = 10): 5 sets of medium-load (67% 1RM) resistance exercise, with 10 repetitions per set, and contractions performed as fast as possible in the concentric phase. The recovery interval between each set was 90 s (3) the CON group (n = 10): the control group was required not to perform any limb strength exercise during the 8-week intervention.

**FIGURE 1:**
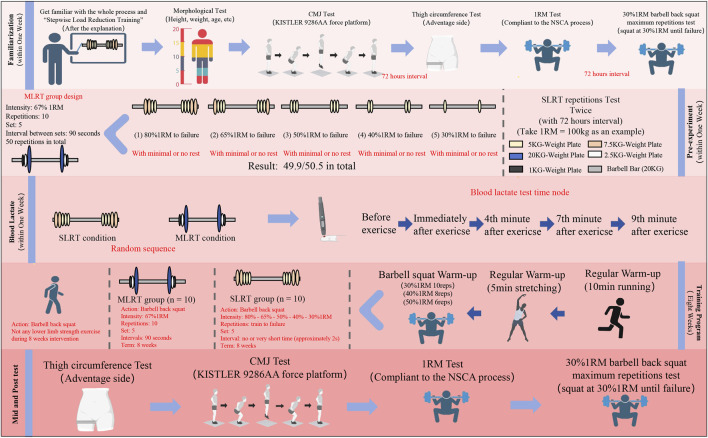
Overview of research design. SLRT: stepwise load reduction training; MLRT: medium load resistance training; CON: control group. The blood lactate test results included the blood lactate value before exercise, the immediate blood lactate value, the 4-min blood lactate value, the 7-min blood lactate value, and the 9-min blood lactate value.

Due to the nature of the intervention (resistance training), participants could easily determine their group allocation based on the specific exercises, intensity, or equipment used, so the blinding was not feasible. Both the SLRT and MLRT groups were required to participate in the intervention twice a week, conducted from 2p.m. to 6p.m. on Tuesday and Friday. The intervention lasted for 8 weeks. During the 8-week intervention, participants’ attendance was recorded at each session, and multiple research staff with CSCS certification supervised the participants throughout the training. To accommodate participants who could not attend the scheduled training on Tuesdays and Fridays, alternative training days were offered. The first training of the week could be rescheduled to Monday or Wednesday, and the second training could be adjusted to Thursday or Saturday. This approach ensured that participants could still adhere to the intervention frequency, even if they faced scheduling conflicts, thereby maintaining adherence to the intervention protocol. After 4 weeks of intervention, the barbell back squat 1RM, thigh circumference, and 30% 1RM maximum repetitions were reevaluated in the fifth week as the mid-test. The 30% 1RM maximum repetition test was also conducted after a 72-h washout period. After the last 4-week training session, the post-test was performed in the 10th week. The research design program is shown in Figure 1.

To minimize the potential confounding effects of diet and supplementation, participants were instructed to maintain their usual dietary habits throughout the intervention period. They were explicitly advised not to increase the intake of any specific nutrients or use dietary supplements. Additionally, participants were asked to refrain from engaging in lower-limb strength training but were otherwise allowed to continue their usual physical activities. Although no detailed dietary control was implemented, a basic record of participants’ diet was kept through consultations at each visit, ensuring that no significant changes occurred during the study period.

### 2.7 Statistical analyses

The data were analysed via SPSS 27.0. The normality of the data was confirmed via the Shapiro‒Wilk test, and the results are reported as the means ± standard deviations (M ± SDs). One-way repeated-measures ANOVA was used to examine the differences among the pre, mid-, and posttest data within each group. One-way ANOVA was used to examine differences among the three groups in terms of the 1RM, thigh circumference, 30% 1RM maximum repetitions and CMJ performance. Two-way repeated-measures ANOVA was used to examine the differences in BL. When the main effects were detected, the Bonferroni correction was used to identify the specific differences between each group. The partial η2 was employed to assess the effect size (ES), with the effect size interpreted as medium (>0.50) or large (>0.80). The significance level was set at *p* < 0.05. The intraclass correlation coefficient (ICC) was used to ensure the reliability of all tests. When the ICC was ≥0.75, it met the established “high reliability” measurement standard (Cicchetti, 1994).

## 3 Results

No significant differences between groups were observed in any baseline values, and all participants completed the study. The detailed results are presented below.

### 3.1 Reliability of measurements

**FIGURE 2:**
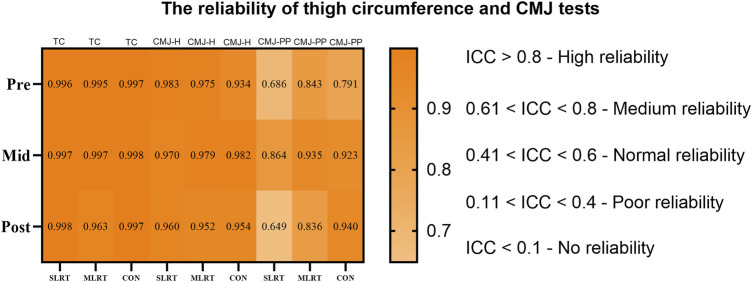
The reliability of thigh circumference and CMJ tests. *NOTE: TC* = thigh circumference, *CMJ - H* = counter movement jump height, *CMJ - PP* = counter movement jump peak power.

To ensure the reliability of the thigh circumference and CMJ tests, intraclass correlation coefficients (ICCs) were calculated for each group at different time points. The results revealed that all groups had ICC values greater than 0.75 for thigh circumference, meeting the standard for “high reliability”. Moreover, all the groups had ICC values greater than 0.60 for the CMJ height and peak power, meeting the standard for “medium reliability”. The detailed results are shown in Figure 2.

### 3.2 Blood lactate

As shown in Table 3, no significant group*time interactions were observed at the five time points except before the test value was reached (*F* = 0.222, *p* = 0.848, *ES* = 0.004). Nevertheless, there was a main effect of time (*F* = 161.949, *p <* 0.001, *ES* = 0.736) and a main effect of group (*F* = 11.144, *p* = 0.0015, *ES* = 0.161).

**TABLE 3:** Time main effect, group main effect and time*group interaction effect in the test of within-subjects effects.

Variables	Within groups								
	Time			Group			Time*group		
	*F* value	*P* value	*ES*-value	*F* value	*P* value	*ES*-value	*F* value	*P* value	*ES*-value
Blood lactate	161.949	<0.001	0.736	11.144	0.0015	0.161	0.222	0.848	0.004

*NOTE: p* < 0.05 indicates a significant difference; *p* < 0.01 indicates a very significant difference.

**FIGURE 3:**
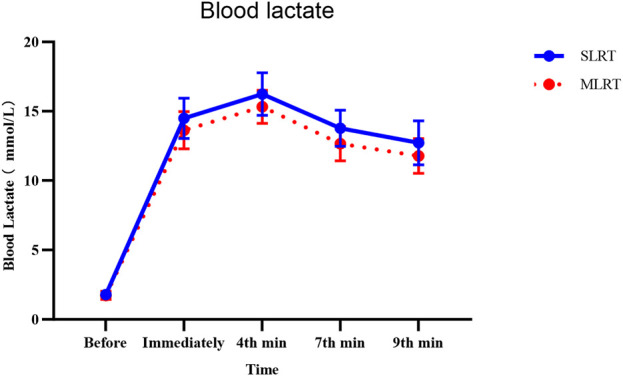
Effects of SLRT and MLRT on the blood lactate concentration (mmol/L) at various time points. The SLRT group presented a more pronounced increase in blood lactate levels post exercise, peaking at the 4th minute and gradually declining thereafter.

Thus, according to the time main effect, we observed that there were very significant differences between any time node value and other time node values independently in both the SLRT group and the SLRT group. According to the group main effect, we observed that there was no significant difference between the SLRT and MLRT groups (1.8 ± 0.3 vs. 1.7 ± 0.3 mmol/L, *p* = 0.52) in blood lactate before the test, and there were significant differences in immediately, 4^th^, 7^th^, and 9^th^ minute blood lactate levels (immediately: 14.5 ± 1.4 vs. 13.6 ± 1.3 mmol/L, *p* = 0.02, *ES* = 0.666; 4^th^: 16.2 ± 1.5 vs. 15.3 ± 1.2 mmol/L, *p* = 0.012, *ES* = 0.663; 7^th^: 13.8 ± 1.3 vs. 12.7 ± 1.2 mmol/L, *p* = 0.001, *ES* = 0.879; 9^th^: 12.7 ± 1.6 vs. 11.8 ± 1.3 mmol/L, *p* = 0.013, *ES* = 0.617). Additionally, to clarify the relationship between peak blood lactate and lactate threshold, we compared the relative peak blood lactate values between the SLRT and MLRT groups, and a significant difference was observed (4.07 ± 0.38 vs. 3.84 ± 0.29 mmol/L, *p* = 0.034, *ES* = 0.406). The relative peak blood lactate value was calculated by dividing peak blood lactate by lactate threshold. Considering that all participants in this study were healthy adult males, without involving more complex populations, and based on existing research, the lactate threshold for healthy adult males is generally around 4 mmol/L (Heck et al., 1985; Faude et al., 2009; Heuberger et al., 2018). Thus, we adopted 4 mmol/L as the lactate threshold for analysis in this study. The detailed data are presented in Table 4, and the change trend of the BL is shown in Figure 3.

**TABLE 4:** Effects of different exercise interventions on changes in blood lactate parameters.

Time nodes	SLRT	MLRT	*p* value
Before	1.8 ± 0.3	1.7 ± 0.3	0.52
Immediately	14.5 ± 1.4^aa^	13.6 ± 1.3^aa^	0.02
4th minute	16.2 ± 1.5^aabb^	15.3 ± 1.2^aabb^	0.012
7th minute	13.8 ± 1.3^aabcc^	12.7 ± 1.2^aabbcc^	0.001
9th minute	12.7 ± 1.6^aabbccdd^	11.8 ± 1.3^aabbccdd^	0.013
Relative peak blood lactate	4.07 ± 0.38	3.84 ± 0.29	0.034

*NOTE:* an indicates a significant difference compared with the before value, *p* < 0.05.

^b^ Indicates a significant difference compared with the immediate value, *p* < 0.05.

^c^ Indicates a significant difference compared with the 4^th^ min value, *p* < 0.05.

^d^ Indicates a significant difference compared with the 7^th^ min value, *p* < 0.05; double characters indicate very significant differences, *p* < 0.01; *p* < 0.05 indicates a significant difference; *p* < 0.01 indicates a very significant difference.

### 3.3 One-repetition maximum barbell back squat

As shown in Table 5, the SLRT group significantly increased from Pre to Mid, from Mid to Post and from Pre to Post (130.4 ± 6.7 kg vs. 136.9 ± 4.7 kg vs. 142.6 ± 6.3 kg, *p* < 0.01, *F* = 102.56, *ES* = 0.919), and the MLRT group significantly increased from Pre to Mid, from Mid to Post and from Pre to Post (129.1 ± 7.0 kg vs. 134.5 ± 6.4 kg vs. 137.9 ± 8.6 kg, *p* < 0.01, *F* = 37.39, *ES* = 0.806). The Con group significantly decreased from Pre to Mid, from Mid to Post and from Pre to Post (128.6 ± 6.4 kg vs. 125.5 ± 6.6 kg vs. 122.5 ± 6.7 kg, *p* < 0.01, *F* = 100.88, *ES* = 0.918).

**TABLE 5:** Effect of different exercise interventions on the change in strength performance parameters.

Variables	Group	Pre	Mid	Post	*F*_*1*_	*ES*_*1*_	Variation 1	Variation 2
One repetition maximum back squat (kg)	SLRT	130.4 ± 6.7	136.9 ± 4.7^**^	142.6 ± 6.3^**^	102.56	0.919	6.50 ± 2.92^b^ ^,^ ^b^	12.20 ± 2.78^a^ ^,^ ^b^ ^,^ ^b^
	MLRT	129.1 ± 7.0	134.5 ± 6.3^**^	137.9 ± 8.6^**^	37.39	0.806	5.40 ± 1.78^b^ ^,^ ^b^	8.80 ± 3.77^b^ ^,^ ^b^
	CON	128.6 ± 6.4	125.5 ± 6.6^**^	122.5 ± 6.7^**^	100.88	0.918	−3.1 ± 1.10	−6.1 ± 1.73
	*F*_*2*_						64.86	154.38
	*ES*_*2*_						0.878	0.945
Thigh circumference (cm)	SLRT	55.2 ± 2.2	56.6 ± 2.7^**^	57.2 ± 2.7^**^	21.77	0.708	1.32 ± 1.1^b^ ^,^ ^b^	1.99 ± 1.22^a^ ^,^ ^b^ ^,^ ^b^
	MLRT	55.0 ± 1.9	56.5 ± 2.0^**^	57.1 ± 1.9^**^	24.19	0.729	1.47 ± 0.88^b^ ^,^ ^b^	2.08 ± 1.10^b^ ^,^ ^b^
	CON	54.0 ± 1.8	53.8 ± 1.9	53.7 ± 1.8	0.089	0.007	−0.19 ± 0.27	−0.34 ± 0.23
	*F*_*2*_						11.84	17.05
	*ES*_*2*_						0.568	0.655
30%1RM back squat maximum repetitions (reps)	SLRT	37.7 ± 7.1	59.5 ± 7.2^**^	75.4 ± 8.1^**^	122.70	0.932	21.8 ± 6.49^b^ ^,^ ^b^	37.7 ± 9.59^a^ ^,^ ^b^ ^,^ ^b^
	MLRT	35.8 ± 7.3	52.3 ± 5.8^**^	63.0 ± 8.3^**^	71.19	0.888	16.50 ± 5.08^b^ ^,^ ^b^	27.2 ± 8.99^b^ ^,^ ^b^
	CON	36.2 ± 5.4	33.1 ± 5.8^*^	30.8 ± 5.8^**^	19.689	0.686	−3.1 ± 3.18	−5.4 ± 3.44
	*F*_*2*_						74.96	78.52
	*ES*_*2*_						0.893	0.897

*NOTE: Variation 1* refers to the difference between the Mid test and the pre test (mean ± SD). *Variation 2* refers to the difference between the post test and the pretest (mean ± SD). * indicates a significant difference compared with the pretest, *p* < 0.05.

^a^ Indicates a significant difference compared with the MLRT, group, *p* < 0.05.

^b^ Indicates a significant difference compared with the Con group, *p* < 0.05; double characters indicate very significant differences, *p* < 0.01.

**FIGURE 4:**
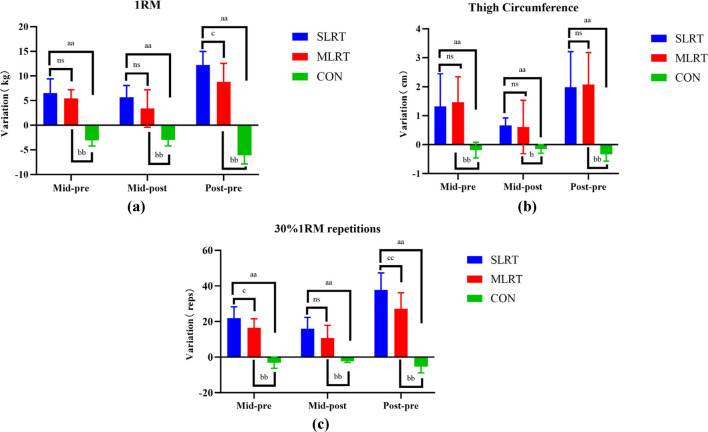
**(a–c)** Changes in maximum strength, thigh circumference and muscle endurance after the 8-week intervention in the three groups. **(a)** represents the change trend of 1rm in three groups, **(b)** represents the change trend of thigh circumference in three groups, **(c)** represents the change trend of 30%1rm repetitions in three groups. **(a)** indicates a significant difference between SLRT and CON, *p* < 0.05; **(b)** indicates a significant difference between MLRT and CON, *p* < 0.01; **(c)** indicates a significant difference between SLRT and MLRT; ns indicates no significant difference, *p* > 0.05. Double characters indicate highly significant differences, *p* < 0.01.

As shown in Figure 4a, there was no significant difference between the SLRT and MLRT at Pre to Mid and Mid to Post (6.5 ± 2.9 kg vs. 5.4 ± 1.8 kg, *p* = 0.352; 5.7 ± 2.4 kg vs. 3.4 ± 3.8 kg, *p* = 0.120). However, after the entire 8-week SLRT intervention, the promotion of the one-repetition maximum barbell back squat was prominently greater than that after the MLRT (12.2 ± 2.8 kg vs. 8.8 ± 3.8 kg, *p* = 0.045, *ES* = 1.018).

### 3.4 Thigh circumference

As shown in Table 5, after 8 weeks of intervention, the SLRT group significantly increased from Pre to Mid, from Mid to Post and from Pre to Post (55.2 ± 2.2 cm vs. 56.6 ± 2.7 cm vs. 57.2 ± 2.7 cm, *p* < 0.01, *F* = 21.77, *ES* = 0.708), and the MLRT group significantly increased from Pre to Mid and from Pre to Post (55.0 ± 1.9 cm vs. 56.5 ± 2.0 cm vs. 57.1 ± 1.9 cm, *p* < 0.01, *F* = 24.19, *ES* = 0.729). However, no difference was observed in MLRT group from Mid to Post (*p* = 0.66). The Con group significantly decreased from Pre to Mid, from Mid to Post and from Pre to Post (54.0 ± 1.8 vs. 53.8 ± 1.9 vs. 53.7 ± 1.8, *p* = 0.915, *F* = 0.089, *ES* = 0.007).

Moreover, as shown in Figure 4b, there was no significant difference between the SLRT and MLRT at Pre to Mid (1.3 ± 1.2 cm vs. 1.5 ± 0.9 cm, *p* = 0.76), Mid to Post (0.7 ± 0.3 cm vs. 0.6 ± 0.9 cm, *p* = 0.845) and Pre to Post (2.0 ± 1.2 cm vs. 2.1 ± 1.1 cm, *p* = 0.88).

### 3.5 Maximum repetitions of 30% 1RM barbell back squat

As shown in Table 5, after 8 weeks of training, the SLRT group clearly improved from Pre to Mid, from Mid to Post and from Pre to Post (37.7 ± 7.1 reps vs. 59.5 ± 7.2 reps vs. 75.4 ± 8.2 reps, *p* < 0.01, *F* = 122.70, *ES* = 0.932), and the MLRT group significantly improved from Pre to Mid, from Mid to Post and from Pre to Post (35.8 ± 7.3 reps vs. 52.3 ± 5.8 reps vs. 63.0 ± 8.3 reps, *p* < 0.01, *F* = 71.19, *ES* = 0.888). The Con group significantly decreased from Pre to Mid, from Mid to Post and from Pre to Post (36.2 ± 5.4 reps vs. 33.1 ± 5.8 reps vs. 30.8 ± 5.8 reps, *p* < 0.01, *F* = 19.689, *ES* = 0.686).

As shown in Figure 4c, there was no significant difference between the SLRT and MLRT at Pre to Mid and Mid to Post (21.8 ± 6.5 reps vs. 16.5 ± 5.1 reps, *p* = 0.059; 15.9 ± 6.4 reps vs. 10.7 ± 7.2 reps, *p* = 0.105), but there was a significant difference between the SLRT and MLRT at Pre to Post (37.7 ± 9.59 reps vs. 27.2 ± 8.99 reps, *p* = 0.047, *ES* = 1.130).

### 3.6 Counter movement jump

**FIGURE 5:**
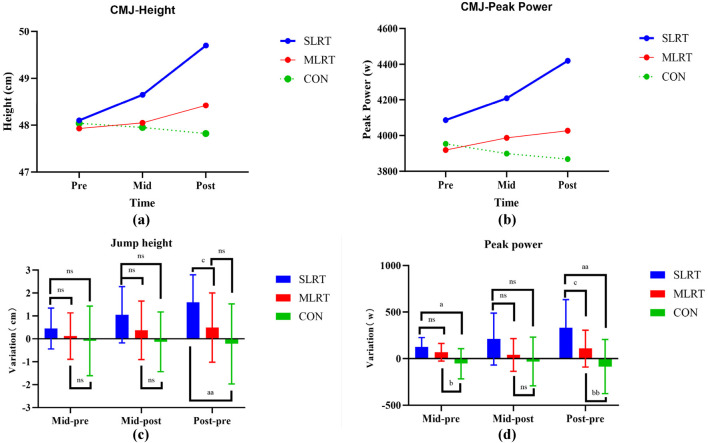
**(a–d)** Changes in counter movement jump height and peak power after the 8-week intervention in the three groups. **(a)** represents the change trend of cmj height in the three groups; **(b)** represents the change trend of cmj peak power in the three groups; **(c)** represents the difference between the two-group comparison in cmj height; **(d)** represents the difference between the two-group comparison in cmj peak power; an indicates a significant difference between SLRT and CON, *p* < 0.05; **(b)** indicates a significant difference between MLRT and CON, *p* < 0.01; **(c)** indicates a significant difference between SLRT and MLRT; ns indicates no significant difference, *p* > 0.05. Double characters indicate highly significant differences, *p* < 0.01.

As shown in Table 6, the jump height and peak power significantly increased after the SLRT from Pre to Mid, from Mid to Post and from Pre to Post (height: 48.1 ± 2.5 cm vs. 48.7 ± 2.3 cm vs. 50 ± 2 cm, *p* < 0.01, *F* = 11.552, *ES* = 0.562; peak power: 4086.4 ± 288.6 w vs. 4209 ± 299.4 w vs. 4419.4 ± 290.2 w, *p* < 0.01, *F* = 9.503, *ES* = 0.514). However, no significant difference in jump height or peak power was detected between the MLRT and CON groups (MLRT height: 47.9 ± 2.4 cm vs. 48.1 ± 2 cm vs. 48.4 ± 2.1 cm, *p* > 0.05; MLRT peak power: 3919.3 ± 226.9 w vs. 3987.3 ± 242.2 w vs. 4026.9 ± 220.5 w, *p* > 0.05; CON height: 48 ± 2.2 cm vs. 48 ± 2 cm vs.47.8 ± 2.1 cm, *p* > 0.05; CON peak power: 3953.7 ± 249.1 w vs. 3898.8 ± 212.4 w vs. 3868.5 ± 275.8 w, *p* > 0.05). The change trends are shown in Figures 5a,b.

**TABLE 6:** Effects of different exercise interventions on changes in cmj performance parameters.

Variables	Group	Pre	Mid	Post	*F*_*1*_	*ES*_*1*_	Variation 1	Variation 2
CMJ - H (cm)	SLRT	48.1 ± 2.5	48.7 ± 2.3	50 ± 2**	11.552	0.562	0.3 ± 0.7	1.6 ± 1.2^a^ ^,^ ^b^ ^,^ ^b^
	MLRT	47.9 ± 2.4	48.1 ± 2	48.4 ± 2.1	0.791	0.081	0.1 ± 1	0.5 ± 1.5
	CON	48 ± 2.2	48 ± 2	47.8 ± 2.1	0.104	0.011	−0.1 ± 1.5	−0.2 ± 1.8
	*F*_*2*_						1.038	10.914
	*ES*_*2*_						0.103	0.548
CMJ - PP (w)	SLRT	4086.4 ± 288.6	4209 ± 299.4*	4419.4 ± 290.2*	9.503	0.514	122.6 ± 103.3^b^	333.1 ± 300.6^a^ ^,^ ^b^ ^,^ ^b^
	MLRT	3919.3 ± 226.9	3987.3 ± 242.2	4026.9 ± 220.5	2.245	0.200	68 ± 95.2^b^	107.6 ± 198.6^b^ ^,^ ^b^
	CON	3953.7 ± 249.1	3898.8 ± 212.4	3868.5 ± 275.8	0.625	0.065	−54.8 ± 161.2	−85.1 ± 290.2
	*F*_*2*_						21.145	21.62
	*ES*_*2*_						0.701	0.706

*NOTE: CMJ - H*, counter movement jump height; *CMJ - PP*, counter movement jump peak power. *Variation 1*refers to the difference between the Mid test and the pretest (mean ± SD). *Variation 2*refers to the difference between the post test and the pretest (mean ± SD).* indicates a significant difference compared with the pretest, *p*< 0.05.

^a^ Indicates a significant difference compared with the MLRT, group, *p*< 0.05.

^b^ Indicates a significant difference compared with the Con group, *p*< 0.05; double characters indicate very significant differences, *p*< 0.01.

As shown in Figures 5c,d, in terms of jump height variation, there was no significant difference between the SLRT and MLRT from Pre to Mid and Mid to Post (0.45 ± 0.9 cm vs. 0.1 ± 1 cm, *p* = 0.327; 1.05 ± 1.23 cm vs. 0.37 ± 1.28 cm, *p* = 0.241), but there was a significant difference between the SLRT and MLRT from Pre to Post (1.6 ± 1.2 cm vs. 0.5 ± 1.5 cm, *p* = 0.034, *ES* = 0.810). Additionally, in terms of peak power variation, there was no significant difference between the SLRT and MLRT from Pre to Mid and Mid to Post (122.6 ± 103.3 w vs. 68 ± 95.2 w, *p* = 0.071; 210.43 ± 279.5 w vs. 39.58 ± 175.14 w, *p* = 0.052), but there was a significant difference between the SLRT and MLRT from Pre to Post (333.1 ± 300.6 w vs. 107.6 ± 198.6 w, *p* = 0.038, *ES* = 0.885).

## 4 Discussion

This study aimed to investigate the effects of SLRT versus MLRT on lower limb muscle strength and BL levels. These two training methods represent different training strategies: SLRT involves a progressive reduction from high to low loads, aiming for comprehensive muscular activation, whereas MLRT maintains a consistent moderate load, emphasizing balanced development of strength and endurance. Although both approaches could theoretically improve muscular strength and metabolic responses, limited empirical evidence exists regarding their comparative efficacy.

### 4.1 Effects on maximum muscle strength performance

The results showed that both SLRT and MLRT significantly improved lower limb strength, with significant differences observed between the first and second halves of the training period. This indicated that both training methods were capable of inducing rapid adaptive responses in the early phase, as well as maintaining progressive stimuli in the later phase to further promote strength improvements. However, SLRT demonstrated superior outcomes and yielded a greater increase in the 1RM than did MLRT. This may be attributed to rapid load transitions in SLRTs, which recruit a broader range of muscle fibres, particularly Type II fibres. From a neuromuscular perspective, the observed improvement in strength can be attributed to the size principle of motor unit recruitment. During resistance training, low-threshold type I muscle fibres are first recruited, followed by high-threshold type II muscle fibres. However, the SLRT can immediately mobilize more type II fibres to participate through the start of high loading, thus resulting in a stronger stimulatory effect on the force output ability. Second, the “reduction” design of the SLRT load structure can extend the time under tension (TUT) of the target muscle group, prolonging the duration of mechanical tension and metabolic pressure, thus promoting muscle protein synthesis and neural adaptation (Atherton and Smith, 2012). The three primary factors of muscle growth—mechanical tension, muscle damage, and metabolic stress—have been identified in current research (Schoenfeld, 2011; Schoenfeld, 2013). Both high mechanical tension and high metabolic stress can activate the mTORC1 signaling pathway, promoting muscle adaptation. The former activates mTORC1 through high-load stimulation (Behringer et al., 2025), while the latter, through the accumulation of metabolites such as lactate, leads to cell swelling. This swelling activates the osmotic pressure sensor, which in turn activates mTORC1, promotes muscle protein synthesis, promotes type II fiber hypertrophy, and increases muscle strength. Moreover, the cell swelling induced by high metabolic stress also affects the mechanosensitive system in muscle cells, potentially promoting the phenotypic transformation of muscle fibers from Type I to Type II. This could be one of the reasons why SLRT enhances force output (Bourdeau Julien et al., 2018). In addition, SLRT may also produce more effective activation in the central nervous system. In continuous training with no or very short intervals (such as 3 s), the motor control mechanism of the cerebral cortex needs to maintain a highly excited state to complete the rapid conversion from high load to low load. This frequent nerve mobilization promotes the efficiency of nerve drive, thereby improving the coordination and quality of force output.

### 4.2 Effects of thigh circumference performance

Both the SLRT and MLRT significantly improved thigh circumference after 8 weeks of intervention. However, a distinguishing feature was that SLRT induced significant changes in both the first and last 4 weeks of the intervention, whereas MLRT did not show significant changes in the last 4 weeks. This suggests that SLRT can rapidly trigger an adaptive response in the short term and continuously provide progressive stimulation throughout long-term training, thereby further promoting muscle growth. The superior effect of SLRT may be attributed to its unique load fluctuation design, which activates the mTORC1 pathway through mechanical tension and metabolic stress to promote muscle protein synthesis, particularly the hypertrophy of Type II muscle fibers. Relevant studies indicate that metabolic stress (such as lactate accumulation) not only influences muscle adaptation through cell swelling but may also enhance muscle metabolic adaptation by activating the AMPK signaling pathway (Thomson, 2018). Additionally, high metabolic stress may activate the PGC-1α pathway, promoting mitochondrial biogenesis, thereby enhancing muscle endurance while supporting the hypertrophy and strength increase of Type II fibers (Fernandez-Marcos and Auwerx, 2011). In contrast, MLRT continuously activates Type I muscle fibers through prolonged moderate-load training, resulting in relatively stable metabolic pressure and extended time under tension (TUT), which contributes to the slow accumulation of muscle size (Kraemer et al., 2002).

However, the results showed that although both training methods demonstrated improvements within their respective groups, and the changes observed in the first and last 4 weeks differed, the difference in improvements between the two groups was not statistically significant after 8 weeks of intervention. Considering the relatively short duration of the intervention and the participants’ recreational training level, we speculate that these factors may have limited the ability to detect significant differences in muscle hypertrophy between the two training methods. Additionally, SLRT involves higher loads and more concentrated stimulation, leading to greater fatigue after training, which, if recovery is insufficient, may limit its effect on muscle hypertrophy (MacDougall and Sale, 2014). Therefore, while SLRT is effective in enhancing strength and anaerobic capacity, it may not be optimal for inducing muscle hypertrophy within the given timeframe, particularly in participants with prior training experience. Future studies with longer training periods, less-trained participants, and more focus on hypertrophic outcomes may better capture the differences between SLRT and MLRT.

### 4.3 Effects on muscular endurance performance

This study used 30% 1RM maximum repetitions as the endurance metric. The results demonstrated that both SLRT and MLRT significantly improved muscular endurance, with SLRT yielding a greater increase compared to MLRT. Notably, significant differences were observed between the first and last 4 weeks of training for both methods. This suggests that both SLRT and MLRT not only trigger rapid adaptive responses in the early stages of training but also sustain progressive stimulation throughout the intervention, further enhancing muscular endurance. Thus, these findings show that SLRT not only produces greater improvements in muscle strength but also more effectively enhances muscle endurance.

The SLRT adopts a design of decreasing from a high load to a low load during training so that the target muscle group can continue to work under fatigue. This strategy simulates the situation of continuous muscle activity in hypoxia during endurance training, effectively enhancing the muscle tissue’s tolerance to lactate and metabolic efficiency. Moreover, the accumulation of blood lactate during this process can activate the AMPK signaling pathway, leading to increased fatty acid oxidation and suppressed lipogenesis, thereby promoting energy metabolism transitions and improving muscle endurance, particularly in the anaerobic-aerobic junction area (Campos et al., 2002; Moreira-Pais et al., 2024). From the perspective of the energy system, SLRT relies mainly on the energy supply of the ATP-CP system and glycolysis system in the high-load stage. With decreasing exercise load, the aerobic energy supply and lipid oxidation pathway of muscle gradually increase (Wang et al., 2011). The situation in which the multienergy system simultaneously participates in training helps the body improve the ability to quickly switch the energy supply system according to the sports demand and then improve the muscle endurance level (Wilkinson et al., 2008). However, the load fluctuation of the MLRT during the entire training process is relatively tiny. Although there is stable stimulation, the activation of the energy system is relatively simple, especially the lack of extreme metabolic pressure, resulting in a lack of stimulation for the improvement of muscle endurance.

### 4.4 Effects of counter movement jumps

The results showed that SLRT significantly enhanced CMJ height and peak power during both the first and last 4 weeks of the intervention. Notably, SLRT demonstrated consistent improvements across the entire 8-week period, indicating that the training induced rapid adaptive responses in the early phase and maintained progressive stimulation in the later phase of the intervention. In contrast, no significant difference was observed between the MLRT and CON groups. This finding highlights the potential of SLRT for improving anaerobic ability.

This trend may be attributed to the structure of SLRT, which alternates between high- and low-load training. This regimen effectively stimulates both muscular strength and endurance development. In particular, the high-load phase generates significant mechanical tension, which can activate the mTORC1 signaling pathway, leading to enhanced muscle protein synthesis and promoting the hypertrophy of type II muscle fibers (Bentzinger et al., 2013). The hypertrophy and functional adaptation of type II fibers are closely associated with improvements in explosive power (Hvid et al., 2010; Methenitis et al., 2019). Therefore, participants in the SLRT group are likely to exhibit more pronounced gains in CMJ performance. However, the MLRT primarily enhances muscular strength and endurance through medium and stable load patterns. While this approach contributes to improvements in both strength and endurance, its effect on activating the mTORC1 signaling pathway may be less pronounced than SLRT, resulting in a more limited improvement in explosive power. In particular, the MLRT is less efficient at stimulating fast muscle fibers, which play a crucial role in explosive movements (Costa et al., 2021). Consequently, the gains in explosive strength observed in the MLRT group may be slightly lower than those achieved by the SLRT group.

### 4.5 Effects on the blood lactate value

Lactic acid is a metabolic product of glycolysis for the body’s energy supply and is rapidly dissociated into lactate and protons (Robergs et al., 2004). The body contains a small amount of lactate at rest, whereas during high-intensity exercise or cardiorespiratory dysfunction, the body is in a state of relative hypoxia, and lactate concentrations increase dramatically (Åstrand et al., 1963; Brooks, 2018; Mungan et al., 2018). The accumulation of lactate during intense exercise lowers the pH of body fluids, impairs energy supply through glycolytic metabolism, inhibits muscle fiber sensitivity to calcium ions, and hinders muscle contraction. This disruption of the internal environment interferes with normal metabolism, induces fatigue, and significantly impairs the athlete’s physical performance. Lactate accumulation not only causes physiological fatigue but may also increase perceived effort, making athletes feel more fatigued during exercise at the same intensity, thereby affecting performance (Wada et al., 2006; Facey et al., 2013; Cairns and Lindinger, 2025).

In this study, blood lactate levels were measured before exercise and at 0, 4, 7, and 9 min after SLRT and MLRT interventions. The results showed that lactate levels were significantly higher in the SLRT group compared to the MLRT group at all time points. Notably, the absolute peak blood lactate in the SLRT group exceeded 16 mmol/L, and the relative peak was over four times the lactate threshold (based on 4 mmol/L), indicating that SLRT induced a much higher metabolic load, resulting in extreme lactate accumulation. First, there was almost no rest between the sets in SLRT, especially after high-load training followed by low-load training, and the muscle continued to contract without sufficient recovery, resulting in dramatic activation of the glycolytic system. This process uses lactic acid as a byproduct, resulting in rapid accumulation of blood lactic acid in a short time (Volek et al., 2016). In contrast, the 90 s interval between MLRT sets helps buffer metabolite accumulation and reduce the peak blood lactate value (hong and Byung-Kwan, 2021). Secondly, in terms of the lactate clearance pathway, blood lactate can be gradually metabolized through hepatic gluconeogenesis, direct oxidation of the myocardium, and skeletal muscle recovery after exercise (hong and Byung-Kwan, 2021). Due to the higher lactate peak and increased lactate clearance pressure, the lactate levels in the SLRT group remained high at 7–9 min after exercise. These findings suggest that SLRT induces greater fatigue and extends recovery time compared to MLRT, and the lactate-induced muscle acidification impairs muscle contraction, leading to a temporary decline in performance.

However, Cairns et al. (2006) systematically discussed the benefits and drawbacks of blood lactate accumulation during exercise, pointing out that while elevated lactate levels may have a negative effect in the short term, lactate should not be viewed solely as a factor impairing performance. In fact, lactate can improve muscle performance during high-intensity exercise. Therefore, athletes and coaches should recognize both the negative and positive effects of lactate and fully understand its role in performance enhancement. In this context, an increased capacity for lactate accumulation is often regarded as an indicator of enhanced anaerobic capacity. Lactate, as a “metabolic signaling molecule,” activates several molecular mechanisms, thereby promoting muscular adaptations (Abdessemed et al., 1999). Firstly, lactate is believed to inhibit the activity of the TSC1/2 complex, indirectly activating the mTOR signaling pathway. This process significantly promotes muscle protein synthesis, which in turn drives muscle hypertrophy and strength gains (Shirai et al., 2022). This mechanism is closely associated with the elevated lactate levels induced by SLRT, leading to muscle hypertrophy and enhanced strength. Secondly, lactate can activate the LKB1 (Liver Kinase B1) pathway, promoting the phosphorylation and activation of AMPK. Once activated, AMPK increases the expression of PGC-1α, which stimulates mitochondrial biogenesis and enhances cellular energy metabolism, thereby improving exercise endurance (Torma et al., 2019). Additionally, lactate can modulate the HIF-1α pathway to activate the vascular endothelial growth factor (VEGF) signaling pathway, promoting the proliferation and migration of endothelial cells, facilitating new blood vessel formation, and enhancing oxygen supply to muscles, thus accelerating recovery (Liu et al., 2024).

Therefore, although the high blood lactate accumulation caused by SLRT may induce more fatigue in the short term, long-term training adaptations may lead to better training outcomes, enhancing endurance and performance.

### 4.6 Limitations

This study has some limitations that need to be considered. Firstly, the sample was limited to healthy, physically active young males, which limits the generalizability of the findings to other populations, such as females, older adults, and individuals with chronic conditions. Secondly, the 8-week intervention period, while sufficient for strength and endurance improvements, may not have been long enough to fully assess the effects on muscle hypertrophy. Thirdly, the high intensity and short rest intervals of SLRT may not be suitable for individuals with lower recovery capacities, such as older adults or clinical populations. Moreover, SLRT requires equipment that allows rapid changes in resistance, which may not be available in all settings. Therefore, further investigation is needed to explore how SLRT can be adjusted to accommodate these groups, ensuring safety and effectiveness. Additionally, this study did not incorporate personalized lactate threshold monitoring. Lactate threshold is an important indicator of exercise intensity and individual recovery capacity. Monitoring lactate threshold could allow for more precise adjustments in training loads, optimizing the intervention’s efficacy. Finally, although the potential biological mechanisms of SLRT were discussed in-depth in the discussion section, based on the current indicators and prior research, this study did not directly measure relevant biological markers, such as signaling pathways or molecular mechanisms. Thus, the mechanistic explanations are still based on inferences.

## 5 Conclusion

This study demonstrated that both the SLRT and MLRT significantly enhanced lower limb muscle strength, hypertrophy, and endurance in healthy, physically active males over an 8-week period. However, SLRT produced superior improvements in maximal strength (1RM), muscle endurance (30% 1RM repetitions), and acute blood lactate response compared with those of MLRT, even though the training volumes were matched. The higher blood lactate accumulation observed following SLRT may reflect a greater anaerobic stimulus and enhanced glycolytic demand, suggesting potential benefits for improving metabolic conditioning and anaerobic capacity. These findings highlight SLRT as a highly effective resistance training strategy, particularly when the goal is to simultaneously increase maximal strength, endurance, and anaerobic performance. However, given that the study was conducted in a sample of healthy, physically active males, the applicability of these findings to other populations, such as females, older adults, or clinical populations, remains unclear.

Future research should aim to investigate the long-term physiological adaptations of SLRT in more diverse populations, including athletes, individuals with lower training status, and clinical populations. These studies would help assess the generalizability of SLRT and its effectiveness across different groups, including sport-specific and rehabilitation contexts. Additionally, mechanistic studies are needed to understand the physiological processes behind the superior effects of SLRT. Exploring how SLRT impacts muscle growth, energy metabolism, and neuromuscular adaptations would provide deeper insights into the mechanisms that drive its benefits. Future studies should also consider incorporating lactate threshold measurements to provide more tailored training protocols, enabling more precise adjustments to exercise intensity and optimizing training outcomes. Furthermore, behavioral adherence in long-term training programs should be examined. Ensuring high adherence rates is essential for achieving sustained benefits. Research should focus on strategies to improve adherence, such as motivation-enhancing interventions and personalized progress tracking, particularly for populations with lower training status or clinical conditions. This would ensure that the effects of SLRT are fully realized over the long term.

## Data Availability

The raw data supporting the conclusions of this article will be made available by the authors, without undue reservation.
